# Spanish juniper gain expansion opportunities by counting on a functionally diverse dispersal assemblage community

**DOI:** 10.1002/ece3.753

**Published:** 2013-09-09

**Authors:** Gema Escribano-Ávila, Beatriz Pías, Virginia Sanz-Pérez, Emilio Virgós, Adrián Escudero, Fernando Valladares

**Affiliations:** 1 Área de Biodiversidad y Conservación, Universidad Rey Juan CarlosC/Tulipán s/n Móstoles, Madrid, Spain; 2 Departamento de Biología Vegetal I (Botánica y Fisiología Vegetal), Universidad Complutense de MadridC/José Antonio Novais, 2, Madrid, Spain; 3 Museo Nacional de Ciencias Naturales CSICC/Serrano 115 dpdo, Madrid, Spain

**Keywords:** Dispersal quality, functional diversity, generalist dispersers, germination, land-use change, old fields, regeneration opportunities, seed size selection, seedling survival, specialized dispersers

## Abstract

Seed dispersal is typically performed by a diverse array of species assemblages with different behavioral and morphological traits which determine dispersal quality (DQ, defined as the probability of recruitment of a dispersed seed). Fate of ecosystems to ongoing environmental changes is critically dependent on dispersal and mainly on DQ in novel scenarios. We assess here the DQ, thus the multiplicative effect of germination and survival probability to the first 3 years of life, for seeds dispersed by several bird species (*Turdus* spp.) and carnivores (*Vulpes vulpes*, *Martes foina*) in mature woodland remnants of Spanish juniper (*Juniperus thurifera)* and old fields which are being colonized by this species. Results showed that DQ was similar in mature woodlands and old fields. Germination rate for seeds dispersed by carnivores (11.5%) and thrushes (9.12%) was similar, however, interacted with microhabitat suitability. Seeds dispersed by carnivores reach the maximum germination rate on shrubs (16%), whereas seeds dispersed by thrushes did on female juniper canopies (15.5) indicating that each group of dispersers performed a directed dispersal. This directional effect was diluted when survival probability was considered: thrushes selected smaller seeds which had higher mortality in the seedling stage (70%) in relation to seedlings dispersed by carnivores (40%). Overall, thrushes resulted low-quality dispersers which provided a probability or recruitment of 2.5%, while a seed dispersed by carnivores had a probability of recruitment of 6.5%. Our findings show that generalist dispersers (i.e., carnivores) can provide a higher probability of recruitment than specialized dispersers (i.e., *Turdus* spp.). However, generalist species are usually opportunistic dispersers as their role as seed dispersers is dependent on the availability of trophic resources and species feeding preferences. As a result, *J. thurifera* dispersal community is composed by two functional groups of dispersers: specialized low-quality but trustworthy dispersers and generalist high-quality but opportunistic dispersers. The maintenance of both, generalist and specialist dispersers, in the dispersal assemblage community assures the dispersal services and increases the opportunities for regeneration and colonization of degraded areas under a land-use change scenario.

## Introduction

Germination and seedling growth and survival are among the most limiting processes in trees regeneration and colonization (Harper 1977). They are closely linked to seed dispersal which provides the basic template on which environmental filters and biotic interactions act to determine recruitment, plant populations’ spatial patterns (Nathan and Muller-Landau 2000), gene flow, and genetic structure (Bacles et al. 2006; Jordano et al. 2007; García and Grivet 2011). Endozoochorous species usually attract a diverse guild of frugivores which generate a complete array of dispersal patterns according to their behavior, morphology, and physiology (Wenny and Levey 1998; Jordano and Schupp 2000; Westcott and Graham 2000; Schupp et al. 2010).

Behavioral traits of frugivores, such as foraging strategies (Chávez-Ramirez and Slack 1994; Morales et al. 2012) and the intense use of particular habitat features (Schupp and Fuentes 1995; Karubian et al. 2010; Rodríguez-Pérez et al. 2011) greatly determine nonrandom deposition patterns in microhabitats across the landscape (Clark et al. 2005; Russo et al. 2006). Morphological animal traits such as gape width (Rey et al. 1997), gut length, and body size are also important for seed dispersal. For instance, bigger body size is related to longer gut retention time which usually promotes longer dispersal (Jordano et al. 2007; Spiegel and Nathan 2007; Figuerola et al. 2010), more clumped deposition patterns (Howe 1989), and low germinability due to seed damage (Traveset 1998; Traveset and Verdú 2002).

Frugivore traits determine the so-called qualitative component of seed dispersal (Schupp et al. 2010) which describes the effectiveness of each disperser in terms of recruitment probability. It has two subcomponents: (i) quality of treatment a seed is given in mouth and gut which influences seed dormancy breakage and germinability; and (ii) quality of seed deposition determined by dispersers’ deposition clumping pattern and microhabitat suitability for seed survival, seed germination, and subsequent survival and growth. Throughout the article we refer to these two components together as dispersal quality (DQ) which is defined as the probability of a dispersed seed generating a new adult.

DQ is usually evaluated in laboratory or green house conditions where only subcomponent (i) is considered (Traveset 1998; Figuerola et al. 2010; Nakashima et al. 2010). Studies dealing with DQ in natural conditions are rare and usually do not account for environmental heterogeneity (microhabitat effect, see Reid 1989). However, subcomponent (i) could interact with several attributes of subcomponent (ii), such as dispersers’ deposition clumping pattern and microhabitat suitability (Howe and Miriti 2004). Therefore, DQ could be highly context dependent and it may vary as environmental conditions change. For instance, Breitbach et al. (2012) show how the dispersal patterns of blackbirds dispersing cherry tree seeds (*Prunus avium*) change with environmental conditions. Blackbirds mobilize seeds further and to more suitable microhabitats in a forest than in a farmland environment. Thus, high-quality dispersers in a well-developed stage of an ecosystem could become low-quality dispersers in disturbed scenarios, such as the blackbirds for the cherry tree, and vice versa. DQ provided by dispersal guilds may shift coupled with environmental changes more frequently than currently recognized.

In the last decade, many studies have focused on the effect of land-use change on biotic interactions. Several studies dealing with the effects of land-use change on mutualistic interactions, such as pollination and seed dispersal, have found a decrease in interaction strength (Tylianakis et al. 2008). Unfortunately, they usually fail or simply do not attempt to investigate the relationship of this weakened effect on the mutualistic interaction and subsequent life stages such as fruit maturation, seed germination, and seedling survival. Therefore, the final outcome of the effect of land-use changes on mutualistic interactions and its derived ecological and evolutionary consequences remains largely unknown (Herrera and Doblas-Miranda 2013). In the case of seed dispersal, we need to establish the link between dispersal guilds and the successive stages which determine plant fitness, thus DQ. This is especially demanding in ecosystems subjected to land-use changes where the DQ provided by each dispersal species or guild could shift under different land-use conditions, which is critical to prevent ecosystem degradation and promote ecosystem recovery opportunities.

In this study we evaluate the DQ of the main dispersal guilds of Spanish juniper (*Juniperus thurifera*), medium-sized birds (*Turdus* spp.), and carnivores (Santos et al. 1999; Escribano-Avila et al. 2012). We also investigate how differential DQ provided by the two assemblages could influence the colonizing and regeneration process after land abandonment in ecosystems dominated by this species. Historically, Spanish juniper has been subjected to traditional management which has been abandoned since the middle of the last century due to population drift. Consequently, *J. thurifera* remnant woodlands are expanding their boundaries and colonizing old fields and grasslands, which are turning into new colonization areas (NCA) (Blanco et al. 2005; Olano et al. 2008). This is a widespread land-use change (Lamb et al. 2005) particularly common in developed countries with remnants populations of junipers (Livingston 1972; Schupp et al. 1997; Rejmének and Rosén 2009).

In a previous work, we found that carnivores contribute more to seed rain in mature woodlands (MW) than thrushes. However, they are opportunist dispersers as this resource is consumed irregularly during the dispersal season (36.5% of feces contained at least one seed), while thrushes present high fidelity to this trophic resource and are considered specialized dispersers (100% of feces contained fruit remains and 60% contained seeds; Escribano-Avila et al. 2012). In recently colonized old fields both carnivores and thrushes contributed similarly to seed rain, although the deposition pattern of each disperser is markedly different. Carnivores preferably disperse seeds in shrubs and open gaps with a highly clumped pattern, while thrushes do under *J. thurifera* trees with one or two seeds per deposition (Escribano-Avila et al. 2012). These microhabitats have previously shown different suitability for the recruitment of the species. *Juniperus thurifera* canopies, especially the female tree, are the most suitable microhabitat while open gaps are the least suitable (Montesinos et al. 2007; Gimeno et al. 2012).

Given the differential dispersal patterns of the two guilds and the different development stages of the ecosystem studied, we hypothesize: (i) thrushes would provide a higher DQ in MW remnants due to enhanced germination (Traveset 1998; Traveset and Verdú 2002) and early survival according to their deposition patterns in more suitable microhabitats (Montesinos et al. 2007). Nevertheless, this enhancement could be limited by the fact that thrushes select smaller fruits due to their gape size limitation (Jordano 1995; Rey et al. 1997; Parciak 2002). As seed size is important in terms of recruitment dynamics (Galetti et al. 2013), smaller seeds could be at a disadvantage especially in less suitable environments such as the recently colonized old fields. Therefore, we hypothesize: (ii) DQ provided by each dispersal guild could shift in the recently colonized old fields in relation to the MWs as the total effect of environmental suitability, seed size, and clumping deposition pattern is poorly understood. To evaluate our hypotheses we provide, for the first time, an evaluation of the probability for a given seed to be recruited accounting for different members on the dispersal community (gut passage effect, clumping pattern, and seed size selection) and environmental heterogeneity under field conditions. To do so, we performed a field germination experiment. We sowed seeds previously dispersed by thrushes and carnivores simulating dispersers’ deposition patterns in the available microhabitats in the two successional stages studied, MWs remnants, and old fields recently colonized by the species.

## Materials and Methods

### Study area

**Figure 1:**
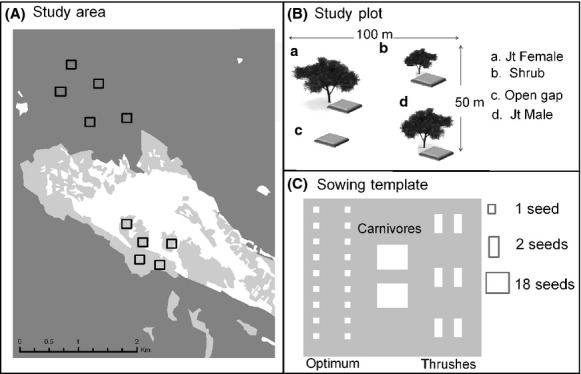
On the left side of the panel, a representation of the study area is shown in (A) to illustrate the ongoing process of woodland expansion. Dark gray represents MWs of *Juniperus thurifera*, light gray represents new colonization areas, and white areas represent current agricultural lands. Squares represent the 50 × 100 plots used to perform the germination experiment. Four microhabitats were selected in each of the plots to cover the environmental heterogeneity in the study area as shown in the top right area of the panel (B). A netting cage was installed in each microhabitat to sow seeds, simulating dispersers’ clumped deposition pattern with the help of a plastic template illustrated in the bottom left-hand (C).

The study area which covers a surface of 13 ha (40°53′N, 2°10′W) is located in the Special Area of Conservation of the Natura 2000 Network Alto Tajo in Guadalajara province, central Spain. The climate is Mediterranean continental with a rainfall of around 500 mm per year with pronounced summer drought and extreme cold winters. Mean annual temperature is 10.2°C, with January being the coldest month (mean temperature: 2.4°C) and July the warmest (mean temperature: 19.5°C). Snowfalls occur from November to April (http://www.aemet.es). Mean elevation is 1278 m, and vegetation is mainly comprised of MWs remnants dominated by *J. thurifera*, old fields recently colonized that are referred to as NCA, and some crop fields (Fig. 1A). MW have a total cover of over 30% with a high abundance of adult trees. Traditional management in these areas has been logging and extensive grazing. NCA comprised pastures where total tree cover is under 15% and most *J. thurifera* individuals are newcomers. Past management in NCA was extensive agriculture and grazing. For more details see Escribano-Avila et al. (2012).

### Seed sampling and presowing treatments

A total of 2640 seeds were sown. We sowed seeds from a control treatment (*n* = 720) and seeds dispersed by two assemblages of dispersers: carnivores composed of red fox (*Vulpes vulpes*) and stone marten (*Martes foina)* (*n* = 1440), and thrushes composed of several species of the genus *Turuds* (*Turdus viscivorus, T. philomelos, T. merula, T. iliacus, T. pilaris*) (*n* = 480), which represent two different functional groups according to their morphological characteristics (gape size, mouth morphology, gut length, and digestive physiology). We assessed the effects of scarification by frugivores on seeds and enhancement on germination rate and survival. The control treatment was used to obtain optimum germination rates for comparison with naturally dispersed seeds, rather than with nondispersed seeds (which have a very low germination rate and could be uninformative [García-Fayos et al. 2001]; M. D. Gargondo, M. A. de Pe\xF1;a, R. de Pedro, N. Verde pers. comm.). This treatment is referred to as the “optimum treatment” throughout the manuscript.

Following García-Fayos et al. (2001), the optimum treatment consisted of seed selection and stratification. Seed selection: fruits with signs of complete maturation (dark blue color) and no signs of parasitization were collected in the study area in the middle of the dispersal season (January 2009) from randomly selected trees. The collected fruits were submerged in water for 2 days. Floating fruits were discarded and for the remaining fruits pulp was removed with a mixer. Viable seeds (no floating) were air dried and sieved to discard seeds with diameters under 3 mm, as smaller seeds have shown very low germination rates. Stratification: control seeds were deposited in trays with sand and water until they reached 70% saturation point and stored at 20°C in darkness for 1 month and at 5°C for an additional month. Seeds dispersed by frugivores were collected in the study area in the same period (for details in dispersed seed collection, see Escribano-Avila et al. 2012). Pellet material from animal depositions was removed, nonviable seeds were removed using the floating method, and once viable seeds were dried, they were sieved in the same way as control seeds. Dispersed and optimum treatment seeds were stored at 4°C until they were sown in the field.

A subsample of nonsieved seeds (*n* = 100) from the optimum treatment and dispersed seeds (by both carnivores and thrushes) were weighed to evaluate the possibility of differential seed size selection by dispersers.

### Field germination experimental design

We selected 10 plots (100 × 50 m). Five were located in MW, whereas the other five were located in NCA (Fig. 1A). We selected four microhabitats in each plot*: J. thurifera* adult female canopy, *J. thurifera* adult male canopy, shrub (*J. communis*), and open gaps. These microhabitats represent the environmental heterogeneity in soil and light exposure variability occurring in the studied ecosystems. We installed a wire netting cage in each microhabitat to avoid seed predation and herbivory (Fig. 1B). Seeds were sown on spring 2009 in different clumping patterns to simulate dispersers’ deposition patterns. Seed clumping (average seeds/deposition) was 1.5 (range 1–5) for thrushes and 73 (range 4–344) for carnivores (Escribano-Avila et al. 2012). According to this information and the quantity of seeds available, we simulated dispersers’ clumped deposition pattern. Seeds were sown in each wire netting cage with the aid of a plastic template. We sowed 18 seeds from the optimum treatment individually in two parallel lines on the left-hand side of the cage, 36 seeds dispersed by carnivores in two groups of 18 seeds in the central area, and 12 seeds dispersed by thrushes in six groups of two seeds on the right-hand side of the cage (Fig. 1C). Cages were monitored periodically for 3 years, and seedling emergence and survival were recorded.

### Data analyses

To evaluate if dispersers perform selection on seed size, we conducted a one-way analysis of variance with seed weight as the response variable and a treatment factor with three levels: thrushes, carnivores, and optimum treatment. Residuals for seed weight fulfilled the assumptions of homoscedasticity and normality.

The variables germination and survival were analyzed with two complementary analyses – generalized linear mixed models (GLMM) and survival analyses – whereas the variable DQ was only analyzed with GLMM. Thus, we performed GLMMs to model germination percentage, survival percentage, and DQ obtained as the percentage of recruited seedlings in relation to total seeds sown. The three variables refer to the end of the 3-year monitoring period. Habitat, disperser, and microhabitat were analyzed as fixed factors, and plot was used as a random factor. We performed model selection on GLMM according to the methodology proposed by Bolker et al. (2009) and Zuur et al. (2009). We first constructed the beyond the optimal model*,* including all fixed effects and their possible interactions (habitat × microhabitat × disperser) and optimized the structure of the random effects (effect of plot on the estimate of the intercept of the model and effect of plot on the estimate of the intercept add up to the parameter estimates of microhabitat). The random structure retained for further analysis was selected by the lowest Akaike information criteria (AIC) and models fitted by restricted maximum likelihood criteria (REML). Once random effects were optimized, we performed model selection for fixed effects fitted by the maximum likelihood criteria (ML). We selected models using the AIC corrected by small sample size, AICc <2 (Burnham and Anderson 2002). When more than one model was selected, we chose which model to be retained based on the Akaike weight (*W*_i_) and the relative importance of the variables in those models (*W*_+_). The final model was fitted by REML to obtain the parameters which better described germination probability, survival, and recruitment. In all cases, error distribution considered was binomial and the link function logit.

Survival analyses were performed to determine the effect of habitat, disperser, and microhabitat on germination rate and the shape of survival curve. We used Kaplan–Meier estimates for right censored data using the log-rank test (Harrington and Fleming 1982). All statistical analyses were conducted in the R environment (R Development Core Team 2012) with additional packages “lme4” (Bates et al. 2012), “MuMIn” (Barton 2012), and “survival” (Therneu 2012).

## Results

### Seed weight

Seed weight for dispersed seeds and the optimum treatment was significantly different (*F*_2_,_297_ = 8.85, *P* < 0.005). Seeds dispersed by thrushes were significantly lighter (0.0279 ± 0.0014) than those dispersed by carnivores (0.0331 ± 0.0014, Bonferroni pairwise test, *P* < 0.005) and those of the optimum treatment (0.03288 ± 0.001, *P* < 0.005). Instead, seeds from the optimum treatment and those dispersed by carnivores did not differ significantly

### Germination, survival, and DQ

**Figure 2:**
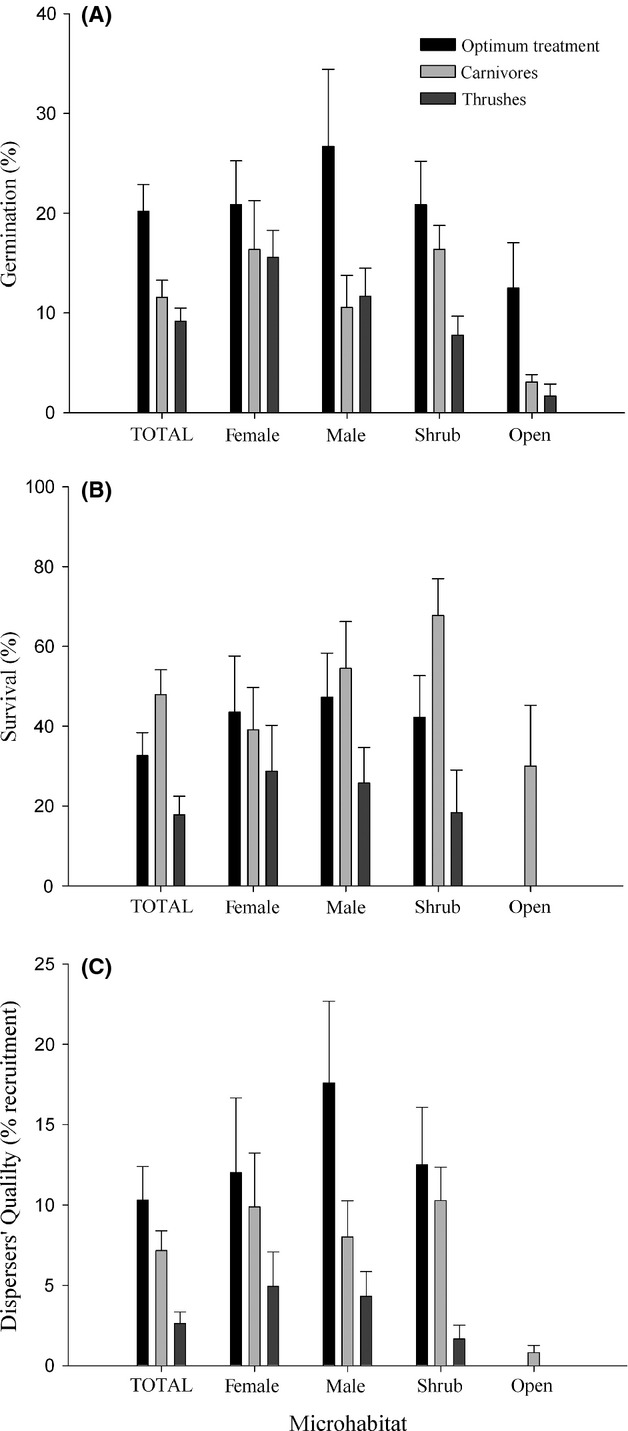
Mean ± SE of the variables germination percentage, survival percentage, and recruitment percentage referred to as dispersal quality in relation to microhabitat and disperser are represented from top to bottom. The first three bars on each graph correspond to the mean percentage of the variable for each disperser and the optimum treatment without distinguishing between microhabitats.

Total germination percentage was 12.5% (*n* = 330), of which 175 seeds germinated in the MW and 155 in NCA. Seeds from the optimum treatment had greater germination percentages than those dispersed by thrushes or carnivores (20% and 10%, respectively). The germination percentage of dispersed seeds was influenced by microhabitat. Greater germination percentages were obtained for seeds dispersed by thrushes beneath female (16%) and male (12%) *J. thurifera* canopies (Fig. 2A), whereas for seeds dispersed by carnivores germination was higher in shrub microhabitats (16.4%). For both dispersers the lowest germination percentages were obtained in the open gap (3% for carnivores and 2% for thrushes). If the effect of dispersers is not considered, the most suitable microhabitat for germination was beneath the *J. thurifera* female tree. This microhabitat accounted for 34% of total germination (*n* = 112), whereas the open gap only accounted for 9% (*n* = 29) (Fig. 2A).

Total survival percentage was 49.33% (*n* = 163), and the MW and NCA had very similar survival percentages (51% and 47%, respectively). Seeds dispersed by carnivores had a notably higher survival percentage (58.6%, *n* = 98) than those dispersed by thrushes (27%, *n* = 18). In terms of microhabitat, the greatest differences in survival percentage were found between open gaps (10.3%, *n* = 3) and all canopied microhabitats (around 50% survival, Fig. 2B).

DQ, measured as the probability of germination and survival during the first 3 years of life, provided by carnivores was higher (6.5%) than that provided by thrushes (2.5%) in all microhabitats (Fig. 2C). Canopied microhabitats provided a higher probability of recruitment (around 15%) than open gaps (1%) where only those seeds dispersed by carnivores were recruited (Fig. 2C). It is noteworthy that DQ provided by carnivores represented 70% of the DQ provided by the optimum treatment when all microhabitats are taken into account, while DQ provided by thrushes only represented 25% (Fig. 2).

### Model selection and parameter estimates

The structure for random effects selected for the germination percentage was plot effect on the intercept of the model and plot effect on the slope of microhabitat (See Table S1), whereas for survival and quality it was plot effect on the intercept (Table S1).

For germination percentage at the end of the 3-year monitoring period, we obtained two models with AICc <2. One included the variables disperser, microhabitat, and disperser × microhabitat, whereas the other model did not include the interaction term. We selected the first model, as the relative importance of the interaction effect was 0.64, while this value was 1 for habitat and microhabitat (Table 1a). Thus, the relative importance of the interaction term was high enough to be included (Burnham and Anderson 2002). The final model included disperser and microhabitat as fully crossed fixed effects and the random effect of plot on the intercept with a standard deviation of 0.81 and the effect of plot on the estimates of the four levels of microhabitat with a standard deviation ranging from 1.13 for *J. thurifera* female trees and 0.54 for *J. thurifera* male trees. (Table 2, random effects).

**Table 1:** Model selection for a. germination, b. survival, and c. disperser quality calculated as the probability of a seed being recruited which implied germination and survival during 3 years

	Models	D	MH	H	D × MH	D × H	H × MH	K	logLik	AICc	Delta	*W*_i_
a. Ger	**1**	**X**	**X**		**X**			**22**	**−90.4**	**235.24**	**0**	**0.64**
	2	X	X					16	−99.57	236.42	1.18	0.36
	*W*_+_	1	1		0.64							
b. Surv	**1**	**X**	**X**					**7**	**−57.33**	**130**	**0**	**0.74**
	2	X	X	X				8	−57.18	132.11	2.1	0.26
	*W*_+_	1	1	0.26								
c. DQ	**1**	**X**	**X**					**8**	**−63.27**	**141.89**	**0**	**0.64**
	2	X	X	X				9	−62.63	143.03	1.13	0.36
	*W*_+_	1	1	0.36								

D, disperser; H, habitat; MH, microhabitat; K, parameters; *W*_i_, akaike weight of the model; *W*_+_, variables relative importance. The selected model for parameter estimation is in bold. A cross indicates that the variable was present in the model.

**Table 2:** Germination estimates and standard errors for the GLMM on germination percentage as a response variable, disperser and microhabitat as full crossed fixed factors, and plot effect on the estimates of the intercept and microhabitat as random factors

Fixed effects	Estimate	SE
Intercept	−2.165	0.387
Female	0.678	0.516
Male	0.929	0.398
Shrub	0.809	0.460
Carnivore	−1.553	0.421
Thrushes	−2.179	0.656
Female × Carnivores	1.237	0.503
Male × Carnivores	0.318	0.506
Shrub × Carnivores	1.255	0.499
Female × Thrushes	1.797	0.727
Male × Thrushes	1.064	0.731
Shrub × Thrushes	1.032	0.748

Random effects	Variance	SD
Intercept	0.66	0.81
Female	1.27	1.13
Male	0.29	0.54
Shrub	0.77	0.88

Missing estimates on levels, “Open,” and “Optimum treatment” are included on the intercept.

Model selection for survival obtained one model with AICc <2, which included disperser and microhabitat as fixed effects. The next model had an AICc = 2.1 and included the same terms as the selected model plus the effect of habitat. The relative importance of habitat in the two models was 0.26 (Table 1), which compared to the relative importance of disperser and microhabitat was not high enough to be considered (Burnham and Anderson 2002). Therefore, the final model selected accounted for the fixed effects of disperser and microhabitat and the random effects of plot on the intercept of the model with a standard deviation of 0.4 (Table 1b, Table 3).

**Table 3:** Survival estimates and standard errors for the GLMM on survival percentage as a response variable, disperser and microhabitat as fixed factors, and plot effect on the estimate of the intercept as a random factor

Fixed effects	Estimate	SE
Intercept	−2.141	0.651
Female	2.156	0.666
Male	2.616	0.666
Shrub	2.417	0.664
Carnivore	0.240	0.282
Thrushes	−1.194	0.367

Random effects	Variance	SD
Intercept	0.16	0.40

Missing estimates on levels, “Open,” and “Optimum treatment” are included on the intercept.

Model selection for DQ obtained two models with AICc <2. The first one included disperser and microhabitat as fixed effects, whereas the second added the effect of habitat. The effect of habitat, as in the case of survival, was not relevant enough to be included (Table 1c). Hence, the final model accounted for the fixed effects of disperser and microhabitat and the random effects of plot on the intercept of the model with a standard deviation of 0.23 (Table 4).

**Table 4:** Disperser quality (DQ) estimates and standard errors for the GLMM on recruitment percentage obtained for the total number of seeds sown and seedling survival after 3 years as response variable, disperser and microhabitat as fixed factors, and plot effect on the estimate of the intercept as a random factor

Fixed effects	Estimate	SE
Intercept	−4.48	0.6
Carnivore	−0.5	0.19
Thrushes	−1.5	0.29
Female	2.74	0.6
Male	2.92	0.6
Shrub	2.73	0.6

Random effects	Variance	SD
Plot (intercept)	0.06	0.23

Missing estimates on levels, “Open,” and “Optimum treatment” are included on the intercept.

### Survival analysis

A total of 330 seeds germinated in the monitoring period (1095 days). In the first year 38% of the seeds germinated (*n* = 124), 48% in the second year (*n* = 159), and 14% in the third year (*n* = 47). The germination curve was not affected by habitat type (log-rank *χ*^*2*^ = 0.1, df = 1, *P* = 0.75). Similarly, neither dispersers nor optimum treatment produced any differences in the germination curve (log-rank *χ*^*2*^ = 2.1, df *=* 2, *P* = 0.34). Microhabitat affected germination rate with germination being significantly slower in the open gap (log-rank *χ*^*2*^ = 61.7, df = 3, *P* < 0.0001, See Fig. S1).

The seedling survival curve was not affected by habitat type (log-rank *χ*^*2*^ = 0.2, df = 1, *P* = 0.7). However, microhabitat and disperser significantly affected seedling survival (log-rank *χ*^*2*^ = 34, df = 3, *P* = 0.0001; *χ*^*2*^ = 12.1, df = 2, *P* = 0.0024, respectively). Seedlings in open gaps died faster than in covered microhabitats. Seedlings dispersed by thrushes also died faster than those from the optimum treatment or dispersed by carnivores (Fig. S1)

## Discussion

Land-use may affect plant regeneration by modifying the relative weight of different demographic stages in the final process of recruitment (Gonzalez-Varo et al. 2012). However, this was not the case in our study, and contrary to our hypothesis no differences were found in germination, seedling survival, or probability of recruitment in the studied stages, mature *J. thurifera* woodland, and NCA. This highlights that the regeneration capacity of the species in old fields is not limited at these critical early life stages. These findings may be related to low-intense traditional agriculture management and to these ancient crop fields being interspersed with natural vegetation. Land-use could affect the quality of dispersal provided by different dispersal vectors (Puerta-Piñeiro et al. 2012). According to our results, DQ provided by carnivores was higher than that provided by thrushes and did not shift between woodland and the disturbed NCA. Thus, the mutualistic interaction between *J. thurifera* and carnivores, a generalist group of dispersers, produced more recruitment than the specialized group of thrushes which selected smaller seeds due to gape width limitation. According to previous results on the quantity of seeds dispersed by thrushes and carnivores (Escribano-Avila et al. 2012) and the differential DQ obtained, the dispersal assemblage of *J. thurifera* is formed by two functional groups which offer a different, but complementary service. Carnivores are opportunistic high-quality dispersers, whereas thrushes are faithful but significantly lower quality dispersers. By maintaining both functional groups, *J. thurifera* ensures its dispersal services under a complete array of environmental scenarios at contrasted spatial and timescales (Fleming et al. 1993).

### Disperser effect on germination and microhabitat interaction

Carnivores, compared to thrushes, are expected to have longer periods of gut retention time which is related to a reduced germinability (Murray et al. 1994). Obviously, this tight connection can be modulated by fruit and seed traits (Traveset and Verdú 2002). *Juniperus thurifera* seeds have a tough seed coat and embryos have a strong, long dormancy. As we found no differences in germination percentages between guilds, we can assume that *J. thurifera* seeds do not suffer damage due to longer gut passage time.

The germination probability of seeds dispersed by thrushes reached a maximum under *J. thurifera* adult trees, whereas in the case of carnivores maximum germination probability was obtained in shrub microhabitats, as shown by the interaction effect between microhabitat and disperser on germination. A similar pattern was found for the quantity of dispersed seeds. Thrushes preferably dispersed seeds beneath the canopy of adult *J. thurifera* trees as a result of their feeding behavior, whereas carnivores deposited more seeds in conspicuous shrubs due to territorial and scent-marking behavior (Escribano-Avila et al. 2012). Therefore, both functional groups of frugivores performed nonrandom dispersal in microhabitats, enhancing germination according to gut passage effect and seed selection performed for each guild. Thus, each functional disperser group generated directed dispersal at the stage of germination (Howe and Smallwood 1982; Wenny and Levey 1998; Howe and Miriti 2004), resulting in a complex dispersal mosaic which was lately modified by seedling survival.

### Microhabitat conditions and seed size selection instead of density dependence–determined recruitment

The highest germination and seedling survival rate of Spanish juniper occurred underneath female juniper tree and similar rates were recorded on male junipers and shrubs. This is quite an unexpected result from the Janzen (1970) and Connell (1971) model perspective (JC hereafter), according to which higher rates of mortality are expected beneath the crown of mother trees due to a higher incidence of pathogens and postdispersal predation. We have not detected seed or seedling predation by pathogens, neither by herbivores in the case of seedlings; instead the most important cause of seedling mortality was desiccation (personal observation). Consequently, our results are better explained by the nurse effect of canopies, that is, facilitation (Lloret et al. 2005) than the JC model. In this study seed and seedling predation by vertebrates were avoided by the use of netting cages, and thus, the effect of vertebrates’ natural enemies could not be evaluated. However, a postdispersal seed removal experiment was performed using the same habitats and microhabitats and two different seed-clumped patterns (data not shown, under preparation). We found similar rates of seed removal in all microhabitats and independently of the clumped pattern which make our recruitment estimates and DQ provided by dispersal guilds robust. Additionally, postdispersal removal rates were similar among MWs and NCA in this study site and therefore the colonization process do not seem to be specially limited by postdispersal predation by mice and rabbits (common seed predators in farming lands).

Medium- to large-sized mammals disperse larger seed clumps than small- to medium-sized birds. According to the JC model, the former are expected to suffer higher mortality than the latter due to negative density dependence. Seeds from the optimum treatment had the higher rate of germination, in this case it is not possible to know which of the two components of the treatment, manual depulpation plus stratification or individual sowing, are responsible for the final outcome. However, in the case of seeds dispersed by thrushes and carnivores the clumping deposition (2 vs. 18 seeds, respectively) does not seem to have an effect as seeds from both dispersers reached similar rates of germination. Therefore, for naturally dispersed seeds of Spanish juniper it seems that there is not an effect of clumping pattern on germination. In the case of seedling survival it seems even clearer that our results do not match the JC model as the seedlings which suffered less mortality rates were those of the most clumped pattern, that is, the seeds dispersed by carnivores.

Instead of by the clumping pattern, our results seem to be better explained by an active seed size selection performed by thrushes. Seeds dispersed by carnivores had a higher survival probability than those dispersed by thrushes. We detected that seeds dispersed by thrushes were smaller than those collected at random from trees and those dispersed by carnivores. Therefore, it seems that thrushes actively selected smaller fruits in the available pool size. This has been described elsewhere for this assemblage and seems to be related to gape width constraints (Jordano 1995; Parciak 2002; Rey et al. 2004). Reduced seed size is known to have a detrimental effect on early survival, as larger seeds usually have larger reserve stocks which plants rely on at this early stage (Venable and Brown 1988; Westoby et al. 1996). Galetti et al. (2013) in a recently published article have shown how the nonrandom loss of a subset of frugivores has pervasive effects on plant regeneration dynamics. They studied the evolutionary and demographic consequences of losing the biggest frugivores on the dispersal assemblage community. Similar results could be expectable in the case of *J. thurifera,* if carnivores were depleted from the dispersal assemblage (i.e., predators control) or do not function as legitimate dispersers due to the abundance of more profitable trophic resources. Under this scenario the colonization of old fields by the species are expected to be compromised or at least decelerated.

According to our results, the adequacy of a microhabitat for germination and early survival is dependent on selection, handling, and the gut passage effect suffered by seeds before they arrive at a given microhabitat. This means that the two subcomponents of DQ (i) quality of treatment in mouth and gut and (ii) quality of deposition could be strongly interrelated (See Rey et al. 2004 for similar results on *Olea europaea*). Similarly, García and Grivet (2011) highlighted how the maternal identity of dispersed seeds and their clumping pattern, both determined by dispersers, have been completely overlooked in seed dispersal studies, even though the nonrandom distribution of genotypes of both conspecifics and heterospecifics in the landscape could have a strong influence on demographic, genetic, and evolutionary patterns (García et al. 2009).

### Differential quality and fidelity of the dispersal assemblage: greater diversity provides more regeneration opportunities

Carnivores are a critical element of the dispersal assemblage of many plant species in highly disturbed habitats, as they usually disperse more seeds than other guilds promoting natural ecosystem recovery (López-Bao and González-Varo 2011; Escribano-Avila et al. 2012; Perea et al. 2012). As shown by our results, they provide high-quality dispersal by improving germination and seedling survival. These findings are especially important in open gaps, as the arrival and establishment of the first trees is a critical stage in the process of natural colonization. Carnivores’ dispersal patterns increase population size and enhance connectivity and gene flow across the landscape, which is especially beneficial in low-density populations where isolation could cause inbreeding or inhibit the reproductive success of self-incompatible species due to pollen limitation (Bacles et al. 2006). By dispersing seeds in open gaps, carnivores increase the probability of recruiting isolated trees. This favors animal movement in general, but especially attracts other species of frugivores, such as birds (Herrera and García 2009) producing a synergic effect on seed mobilization (Howe and Miriti 2004). Verdú and García-Fayos (1996) described how this perch effect promotes the colonization of old fields in a nucleated pattern around the isolated trees in a Mediterranean landscape. The dispersal pattern performed by carnivores simulates an active restoration practice based on the plantation of pioneer trees or clumps (i.e., woodland islets) which act as a stepping stone for the activity of a complex assemblage of dispersers in former deforested lands (Lamb et al. 2005; Benayas et al. 2008). This has the outstanding advantage that carnivores do it for free.

However, carnivores are generalist feeders which have the ability to shift their food consumption to different resources depending on the different trade-offs among food profitability, energy, protein content, and the time invested in obtaining such food (Stephens and Krebs 1986; Genovesi et al. 1996; De Marinis and Asprea 2004). As a result, their role as seed dispersers is commonly opportunistic (Herrera 1989; Zhou et al. 2008). In the study area, we detected a decrease in fruit consumption by carnivores in one of our MW and in several NCA, probably as a consequence of the higher local diversity of trees and shrubs which could provide a higher abundance of prey (small mammals and insects) and promote a shift in carnivores’ trophic resource consumption (Escribano-Avila et al. 2012). Therefore, the maintenance of thrushes in the dispersal community, even though they are not high-quality dispersers, provides a reliable dispersal service to the tree and regeneration process as a whole, as they are trustworthy dispersers independent of the context (Escribano-Avila et al. 2012). Maintaining a diverse dispersal community seems to be a successful strategy for the persistence of the species, as *J. thurifera* has overcome several environmental changes throughout its long history since the tertiary (Terrab et al. 2008). Nowadays, the species is clearly benefitting from its diverse dispersal assemblage, given the spectacular transformation of old fields into NCA (Gimeno et al. 2012). The maintenance of diverse dispersal assemblages has been recently related to ecosystem resilience (Garcia and Martinez 2012), especially in cases where different dispersers provide a similar service to their interacting plant species. Recently, this has been referred to as functional redundancy and makes plant populations less vulnerable to the loss of dispersal species (García et al. 2013; Plein et al. 2013). This suggests that the resilience capacity of an ecosystem is dependent not only on the species diversity but also on the link between species diversity and functionality of the dispersal assemblage (Naeem et al. 1994; Jonsson et al. 2002; Pocock et al. 2012). However, on the seed dispersal framework there is no clear definition of what is considered as “functional diversity”. Thus, a precise definition of what is considered as functional diversity for a dispersal assemblage community and a detailed clarification in this sense is necessary. From our point of view, dispersal functionality should include information on the probability of recruitment for dispersed seeds by different members on a dispersal assemblage accounting for natural heterogeneity. In this sense, our work is a good contribution on the understanding of dispersal functionality, although much more empirical studies are needed in order to know the functional diversity of dispersal assemblages and to build a general framework.

## Conclusions

Old fields abandoned due to rural exodus have a strong potential for natural regeneration, if certain perturbation thresholds are not passed (Cramer et al. 2008) and seeds are supplied by the dispersal community. Different guilds of dispersers could provide differential functional services to plant species, as found in this work. Therefore, the diversity of dispersal assemblages should be correctly managed to favor ecosystem regeneration. Dispersed seed characteristics such as size, maternal origin, and clumping patterns are determined by dispersers’ behavior previous to deposition. These seed characteristics modulate the suitability of microhabitat conditions, and consequently affect recruitment and evolutionary patterns. Unfortunately, to our knowledge this has been overlooked in seed dispersal studies. We consider that explicitly including the effects of nonrandom selection performed by dispersers on seed characteristics in the framework of seed dispersal effectiveness (Schupp et al. 2010) could greatly improve our understanding of the effects of seed dispersal in ecological and evolutionary processes.

## Supporting Information

**Table S1.** Random effects optimization.

**Figure S1.** Survival analyses curves.
