# Supplementary material for: Spanish juniper gain expansion opportunities by counting on a functionally diverse dispersal assemblage community
Source: Ecol Evol. 2013 Sep 9;3(11):3751–63. doi: 10.1002/ece3.753 (PMC3810872; doi:10.1002/ece3.753)
Supplement: Supplementary file 1 [file ece30003-3751-SD1.docx]

**Supporting Information**

**Table S1: Random effects Optimization**

| A) Germination | |  |  |  | AIC | Delta AIC |
| --- | --- | --- | --- | --- | --- | --- |
| **habitat x microhabitat x disperser + (microhabitat\|plot)** | | | | | **226.17** | **0** |
| habitat x microhabitat x disperser + disperser+microhabitat\|plot) | | | | | 240.11 | 13.94 |
| habitat x microhabitat x disperser + (1\|plot) | | | |  | 243.13 | 16.96 |
| habitat x microhabitat x disperser + (disperser\|plot) | | | | | 252.11 | 25.94 |
| B) Survival |  |  |  |  |  |  |
| **habitat x microhabitat x disperser + (1\|plot)** | | | |  | **142.84** | **0** |
| habitat x microhabitat x disperser + (disperser\|plot) | | | | | 150.52 | 7.68 |
| habitat x microhabitat x disperser + (microhabitat\|plot) | | | | | 176.57 | 33.73 |
| C) Quality | | |  |  |  |  |
| **habitat x microhabitat x disperser + (1\|plot)** | | | |  | **20.69** | **0** |
| habitat x microhabitat x disperser + (disperser\|plot) | | | | | 26.75 | 6.06 |
| habitat x microhabitat x disperser + (1\|plot) | | | |  | 30.11 | 9.42 |
| habitat x microhabitat x disperser + disperser+microhabitat\|plot) | | | | | 44.15 | 17.4 |

**Table S1:** Optimization of random effects on Generalized Linear Mixed Models. The models are written according to the syntax of the lme4 package of R environment used to analyze the data. Fixed effects full crossed on the left side of the brackets are always constant only random effects contained on the brackets differ between models. The models with random effects selected for further analyses are shown in bold.

**Figure S1: Survival analyses curves**


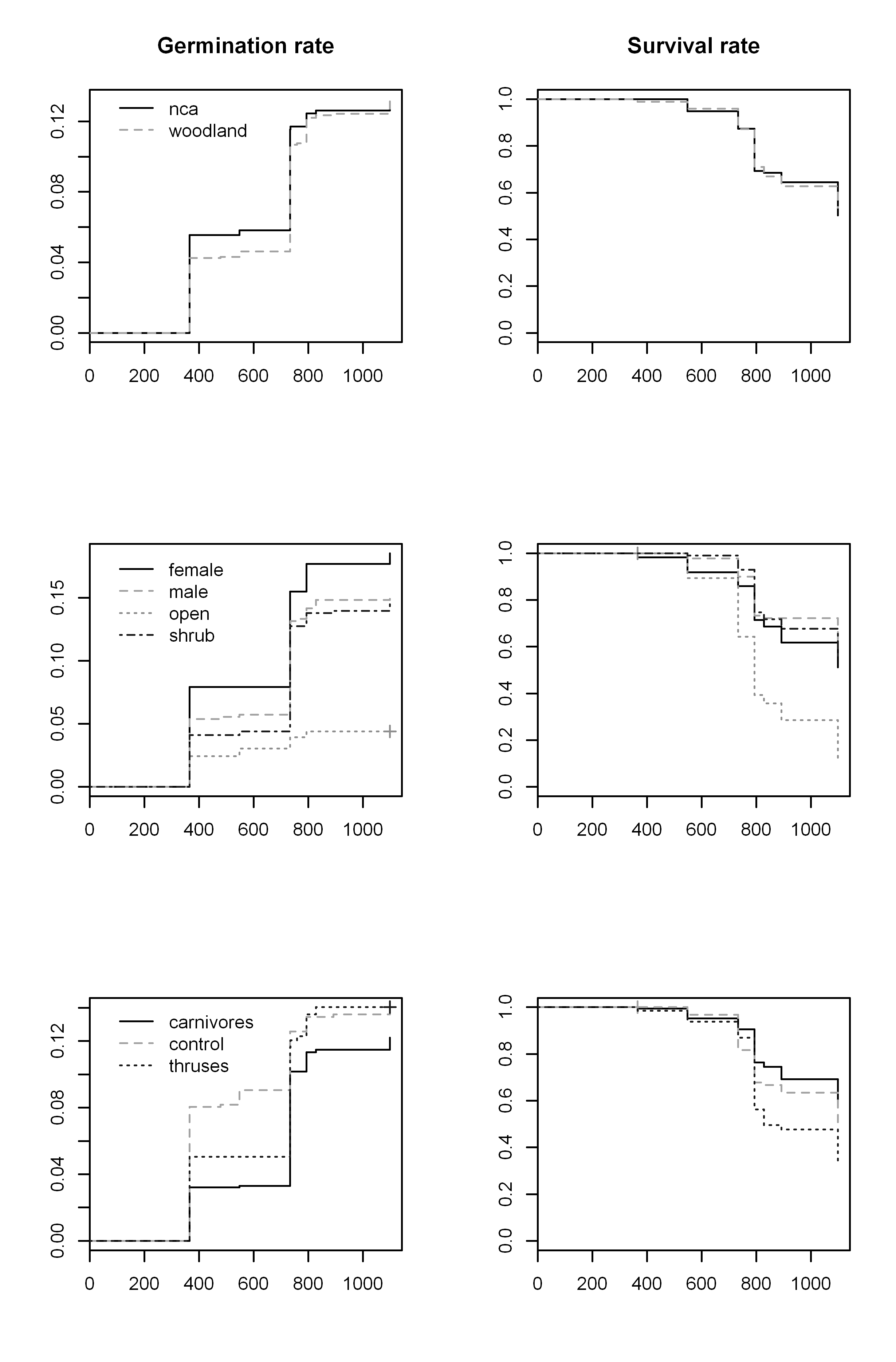


**Fig.S2:** On the left side of the panel germination rates are shown while on survival rates are shown in the right side. From top to bottom: habitat type, microhabitats and dispersers. Time in days from sowing is represented on the horizontal axis. Note the different scale values on the vertical axis. Only microhabitat type had a signficiant effect on germination rate (Kapplan-Meier, log-rank X^2^=61.7 df=3, P<0.0001). Survival rate was significantly affected by microhabitat (X^2^=34 df=3, P=0.0001) and disperser (X^2^=12.1 df=2, P=0.0024).
